# The Management of Lower Extremity Multilevel Arterial Injuries: A 10-Year Experience

**DOI:** 10.1371/journal.pone.0121769

**Published:** 2015-03-20

**Authors:** Hede Yan, Bin Zhao, John Kolkin, Zhijie Li, Xinglong Chen, Tinggang Chu, Weiyang Gao

**Affiliations:** 1 Department of Orthopaedics (Division of Plastic and Hand Surgery), The Second Affiliated Hospital of Wenzhou Medical University, Wenzhou, China; 2 Department of Plastic and Hand Surgery, Duke Raleigh Hospital, Raleigh, North Carolina, United States of America; Azienda Ospedaliero-Universitaria Careggi, ITALY

## Abstract

**Background:**

Limb amputation due to lower extremity arterial injury is not uncommon and multilevel arterial injury is even more limb-threatening and easily missed with potentially devastating consequences. There is limited information on multilevel arterial injuries.

**Purpose:**

We undertook a review of our experience to gain insight on multilevel arterial injury patterns associated with lower extremity trauma and to analyze the results of management of such injuries with a special focus on the influence of initial diagnosis on limb salvage.

**Patients and Methods:**

Between August 2002 and September 2012, 38 patients with lower extremity multilevel arterial injuries were reviewed, retrospectively. The injury patterns and amputation rates associated with initial diagnosis or misdiagnosis were analyzed.

**Results:**

According to their injury levels, three multilevel arterial injury patterns were seen in this series: arterial injuries with the involvement of femoral artery and popliteal artery (pattern A), femoral artery and anterior or (and) posterior artery (pattern B), and popliteal artery and anterior or (and) posterior artery (pattern C). The general missed diagnosis rate was 31.6%. Pattern B had a much higher missed diagnosis rate than the other two patterns. The missed diagnosis rate was significantly correlated with the amputation rates (Odds Ratio =10.7, 95% CI: 2.04-56.61). The definite diagnosis rate was only 14.8% using duplex ultrasonography examination.

**Conclusions:**

Diagnosis of pattern B injury is more prone to be missed. DUS has low specificity in the detection of multilevel arterial injuries. Aggressive intraoperative exploration is considered to be valuable in the definitive diagnosis of highly suspected cases when other diagnostic tools are unavailable.

## Introduction

Lower extremity arterial injuries remain an uncommon but challenging clinical entity, resulting in high rates of lower extremity amputation, functional disability, and mortality, if not treated early and competently.[1,2] The limb salvage rate in patients with simple arterial injuries is over 90%. [3,4] In contrast, complicated vascular injuries, especially in the lower extremity with more tenuous vascular collaterals, may result in much higher amputation rates.[5] Even worse and particularly devastating are multilevel vascular injuries in a mangled extremity. Hafez et al [6] reported a 45% of limb loss rate (Odds Ratio for amputation, 4.4) in patients suffering from above- and below-knee vascular injuries.

Limb amputation in patients with lower extremity arterial injury has been attributed to duration of ischemia, concomitant injuries, development of compartment syndrome, injury mechanism, and failed revascularization.[2,7,8] The reported limb loss and salvage rates vary widely in the literature, partly because of the bias in patient populations and time periods [2]. Nonetheless, the timely and competent management of these devastating injuries is of great importance in terms of their final results.

In practice, timely diagnosis and repair in the treatment of these vascular injuries have been proved to be the key for limb salvage and the restoration of better function.[9–11] Although multilevel vascular injuries are particularly uncommon, they may cause even more devastating consequences with higher limb loss rate and mortality due to the complicated mechanism of injury and the extent of trauma. In particular, the definitive diagnosis of multilevel arterial injuries is sometimes more challenging under emergent situations.

In this retrospective study, we reviewed our 10-year experience with a subset of patients who had lower extremity multilevel arterial injuries. We attempted to summarize and evaluate different vascular injury patterns associated with extremity trauma, focusing on the influence of initial diagnosis on limb salvage.

## Patients and Methods

**Fig 1:**
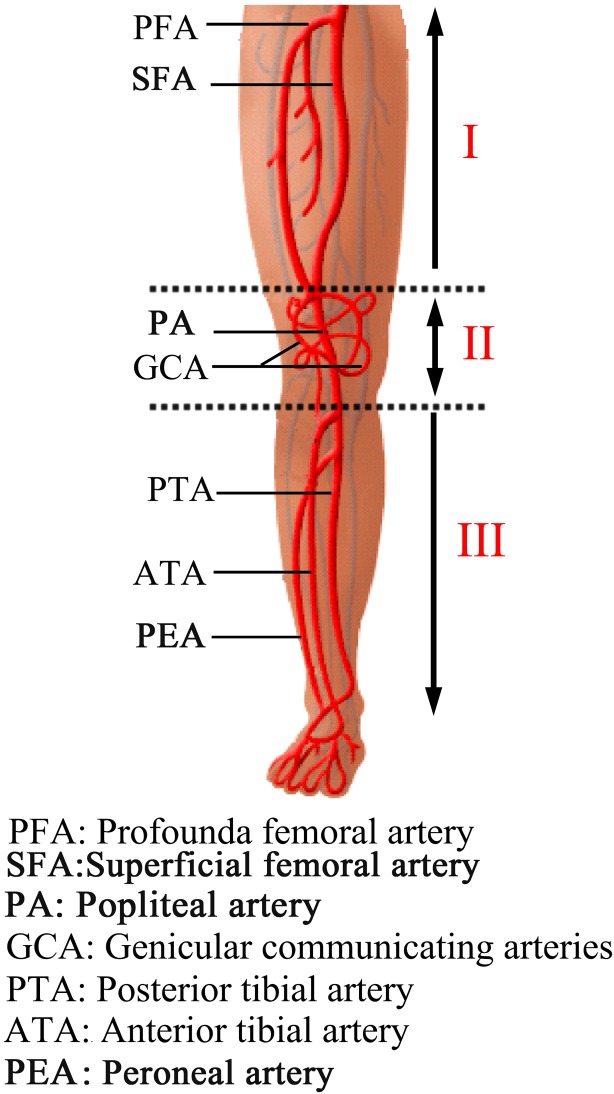
The schematic of injury zones.

Between August 2002 and September 2012, the data of 271 patients suffering from lower extremity arterial injuries treated in our hospital were reviewed retrospectively. For the purposes of this study, only patients suffering multilevel arterial injuries were included and those who sustained single arterial injury or multiple arterial injuries at the same level or underwent primary amputations were excluded. In all, 38 such cases were identified. According to the locations of the arterial injuries, these multiple level injuries were divided into three zones: upper zone injury (femoral artery), middle zone injury (popliteal artery) and lower zone injury (posterior and anterior tibial arteries) (Fig. 1). This study was granted permission by the Research Ethics Committee of the Second Affiliated Hospital of Wenzhou Medical University and all the patient records were anonymized and de-identified prior to analysis. The individual in this manuscript has given written informed consent (as outlined in PLOS consent form) to publish these case details.

Primary diagnosis was usually determined by physical examination, history of trauma and by Doppler ultrasonography in selected patients and the definitive diagnosis was confirmed by the intraoperative findings during exploration. Vascular management was carried out prior to tendon and nerve repair in the operating room under general anesthesia. Bone fractures were treated first in cases with unstable, comminuted fractures using external fixators or some simple fractures, which were estimated to be accomplished within 30 minutes, or else, a temporary arterial shunt was applied. Otherwise, bone immobilization was performed after arterial anastomosis. Systemic heparinization was employed except for arterial injuries with severe soft tissue and muscle injuries. The contralateral saphenous veins were used for interpositional vein grafts, which were used liberally. Venous injuries involved were repaired whenever possible to prevent postoperative venous congestion and to minimize the potential of developing extremity compartment syndrome. At the same time, immediately after revascularization, fasciotomy involving four compartments of the lower legs in all of the patients was carried out using a posteromedial and an anterolateral skin incision either therapeutically or prophylactically. Postoperatively, a protocol of anticoagulation with low-molecular-weight heparin was strictly followed in all the cases and dextran therapy was also given.

### Statistical analysis

The Kolmogorov–Smirnov test was used to analyze the normality of the variables. The independent patterns between groups were compared by One Way ANOVA. The categorical variables were analyzed using Pearson chi-square test and Fisher exact test. The differences between groups were compared using Bonferroni correction. Mean and standard deviation (SD) were used for continuous variables. Statistical significance with a two-sided P value of less than 0.05 was adopted. All the statistical analyses were performed with SPSS v. 19.0.

## Results

Thirty males (78.9%) and 8 females (21.1%) were involved in this series, ranging in age from 17 years to 61 years with a mean age of 31.7 years. Based on the injury patterns, all the patients included were classified into three patterns: pattern A with arterial injuries in the upper and middle zones, pattern B with arterial injuries in the upper and lower zones and pattern C with arterial injuries in the middle and lower zones.

**Fig 2:**
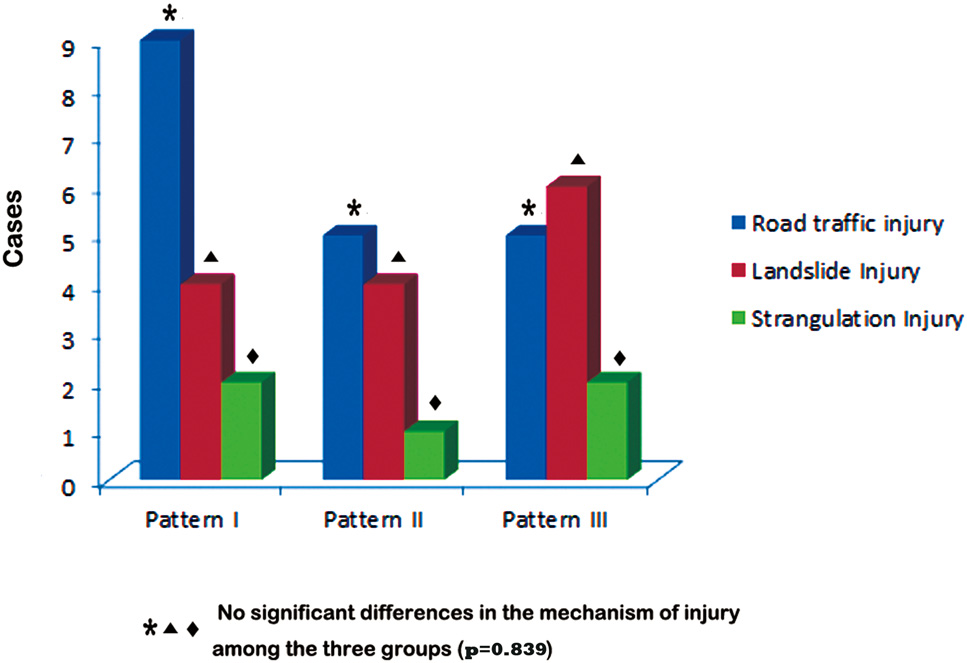
Results of mechanism of injury.

There were 15 cases in pattern A, 10 cases in pattern B and 13 cases in pattern C. Duration of time before surgery ranged from 2 hours to 6 hours with an average time of 3.2 hours. In terms of injury patterns, 18 cases (47.4%) were closed injuries and 20 cases (52.6%) were open injuries. No significant differences in duration of ischemia (p = 0.425) and injury patterns (p = 0.599) were seen among the three patterns. Patients in pattern B suffered from a lower fracture occurrence rate of only 20% in comparison with 86.7% and 76.9% in pattern A and pattern C, respectively (pattern B vs. A, p = 0.002; pattern B vs. pattern C, p = 0.012). The details regarding the patients’ demographics were listed in Table 1. The mechanism of injury was blunt trauma in all the patients: road traffic injury in 55.53% (21/38), landslide trauma in 31.6% (12/38), and strangulation injury by ropes in13.2% (5/38). No significant differences existed between mechanism of injury and injury pattern (p = 0.839) (Fig. 2).

**Table 1:** Demographic data.

Patterns(n)	Zone involved*	Sex(F/M)	Age(mean, years)	Duration of ischemia♦	Injury types^▼^		Concomitant venous injuries^▼^		Long bone fractures^▲^		MESS score♦
					Closed injury (%)	Open injury (%)	Yes	No	Yes	No	
Pattern A(15)	I+II	4/11	17–55(31.9)	2–6(3.0)	7(46.7)	8(53.3)	10(66.7)	5(33.3)	13(86.7)	2(13.3)	6.1(5–7)
Pattern B(10)	I+III	1/9	19–59(29.7)	2–4.5(3.1)	6(66.7)	4(33.3)	7(70)	3(30)	2(20)	8(80)	6.3(4–7)
Pattern C(13)	II+III	3/10	20–61(32.8)	2–5.5(3.5)	5(38.5)	8(61.5)	10(76.9)	3(23.1)	10(76.9)	3(23.1)	5.9(5–7)

* Zone I, femoral artery; Zone II, popliteal artery; Zone III, posterior and anterior tibial artery.

♦ No significant differences in duration of ischemia (p = 0.424) and the MESS scores (Mingled Extremity Severity Score, p = 0.537) among the three patterns

^▼^No significant differences in injury types (Fisher’s exact test, p = 0.599) and the occurrence of concomitant venous injuries (p = 0.903) among the three patterns.

^▲^The fracture occurrence rate in the pattern B was lower than the other two patterns (Fisher’s exact test: pattern A vs. B, p = 0.002; pattern B vs. pattern C, p = 0.012).

Overall, multilevel arterial injuries were initially misdiagnosed as a single level injury in 12 patients (31.6%). All of these patients underwent re-exploration and the definitive diagnosis of multilevel vascular injuries was confirmed during the secondary management. The differences in missed diagnosis rate were significant among the three patterns (p = 0.014), of which pattern B had a much higher missed diagnosis rate (70%) than the other two patterns (20% and 15.4% in pattern A and C, respectively). The amputation rate of pattern B was also much higher than those of the other two patterns (pattern A vs. B, p = 0.028; pattern B vs. pattern C, p = 0.039, respectively) (Table 2).

**Table 2:** Missed diagnosis rates and amputation rates of different patterns.

Patterns	Cases	Missed diagnosis rate (%) ♦	Limb salvage rate (%)	Amputation rate (%) ^▲^
Pattern I	15	3(20)	13(86.7)	2(13.3)
Pattern II	10	7(70)	4(40)	6(60)
Pattern III	13	2(15.4)	11(84.6)	2(15.4)
Total	38	12(31.6)	28(73.7)	10(26.3)

♦Fisher’s exact test showed that the differences in the missed diagnosis rates were significant among the three patterns (p = 0.014);

^▲^The amputation rate in pattern II was much higher than the other two patterns (Fisher’s exact test: pattern I vs. II, p = 0.028; pattern II vs. pattern III, p = 0.039, respectively).

In this series, no patients in this study died. The amputation rate in those patients with an initial diagnosis of multilevel vascular injury was only 11.5% (3/26), while it was significantly higher with 58.3% (7/12) in the misdiagnosed cases (p = 0.005). The correlation between missed diagnosis rate and amputation rate, confidence interval (95% CI), odds ratios (OR), and main findings were listed in Table 3. The missed diagnosis rate was significantly correlated with the amputation rates (OR = 10.7, 95% CI: 2.04–56.61).

**Table 3:** The correlation of initial diagnosis and amputation rate.^▲^

Status of initial diagnosis	cases	Limb salvage rate (%)	Amputation rate (%)
Definite diagnosis	26	23(88.5)	3(11.5)
Missed diagnosis	12	5(41.7)	7(58.3)
Total	38	28(73.7)	10(26.3)

^▲^Chi-square test showed that the amputation rate in patients with missed diagnosis was much higher than those with initial definite diagnosis (p = 0.005, OR = 10.7, 95% CI: 2.04–56.61).

Duplex ultrasonography examination was performed in 27 patients (75%). The definitive diagnosis rate was only 14.8% with a suspicion of injury rate of 18.5% and a 66.7% rate of missed diagnosis. No significant differences in the results of color ultrasonography examination were revealed among the three patterns (p = 0.965) (Table 4).

**Table 4:** Results of duplex ultrasonography examination in diagnosis of multilevel arterial injuries.^▲^

patterns	cases	Detection of vascular injury (%)	Definite diagnosis (%)	Suspect diagnosis (%)	Missed diagnosis (%)
Pattern A	11	11(100)	1(9.1)	2(18.2)	8(72.7)
Pattern B	7	7(100)	1(14.3)	1(14.3)	5(71.4)
Pattern C	9	9(100)	2(22.2)	2(22.2)	5(55.6)
Total	27	27(100)	4(14.8)	5(18.5)	18(66.7)

^▲^Fisher’s exact test showed no significant differences in the results of duplex ultrasonography examination among the three patterns (p = 0.965).

### Case report

**Fig 3:**
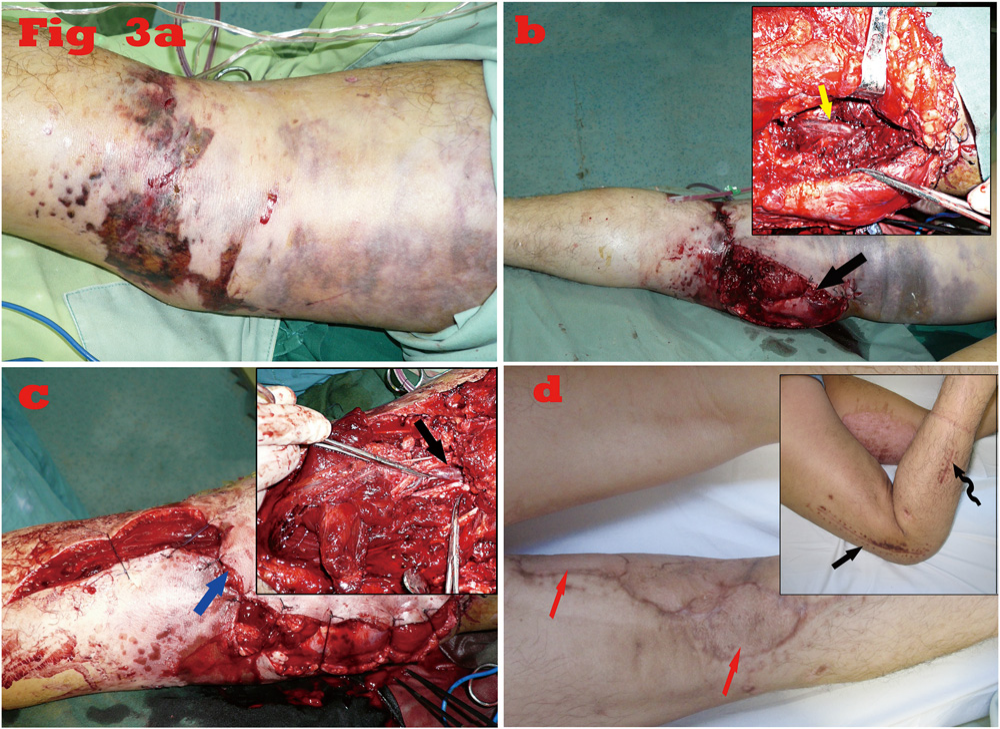
A representative case. a. Preoperative view. The straight arrow indicates the popliteal fossa and the curved one indicates the medial femoral condyle. b. Intraoperative view during primary exploration. The yellow arrow on the top in the inset shows the appearance of the injured femoral artery; the black arrow on the bottom indicates the site of arterial injury. c. Intraoperative view during re-exploration. The black arrow on the top in the inset shows the appearance of the injured popliteal artery; the blue arrow on the bottom indicates the site of arterial injury. d. Postoperative view at 15-month follow-up. The black arrows on the top in the inset show the appearance of lateral incisions for fasciotomy and internal fixation of the femur fracture, respectively; the red arrows on the bottom show the sites of skin grafting for the coverage of wounds.

A 35-year-old man was referred to our hospital 2 hours after a run-over injury to his left leg by a truck. He sustained a closed distal femur fracture with extensive soft tissue injury. The left dorsalis pedis artery was not palpable at admission. The color ultrasonography examination reported a femoral artery injury at above-knee level. Surgical management was soon carried out after necessary preoperative preparation. The femoral artery was contused and thrombosed around the fracture site. The fracture was quickly reduced and immobilized with internal fixation using an AO compression plate. The femoral artery was repaired with an interposition saphenous vein graft from the contralateral side. Four-compartment fasciotomy was performed prophylactically. Four hours after surgery, the skin temperature of the right lower leg remained relatively low and the palpation of dorsalis pedis artery was still uncertain. After routine management of the vascular crisis with no obvious improvement within 30 minutes, re-exploration in the operation room was performed. Surprisingly, the patency of the above-knee anastomosis was confirmed. Further exploration revealed that the popliteal artery was also injured and thrombosed. After repair with an interpositional vein graft, the blood supply of the left leg was resumed and the leg was salvaged. The patient was discharged 15 days after re-exploration (Fig. 3).

## Discussion

The outcomes of the management of lower extremity arterial injuries are time dependent and the duration between injury and restoration of blood supply to the affected limb is of great importance to patients prognosis.[12] Undoubtedly, a prompt and definitive diagnosis of these injures is critical to guiding the development of surgical strategies and reducing limb ischemia time. A majority of simple lower limb arterial injuries can be diagnosed using the combination of a good physical examination and duplex ultrasonography (DUS).[9] It was reported that careful physical examination was highly sensitive and specific in screening vascular injuries in patients with penetrating vascular injuries within extremity and could achieve a negative predictive value of 99% [13] The use of duplex scanning in the diagnosis of such injuries was also reported to have both sensitivity and specificity of more than 90%.[14,15]

However, the diagnosis of blunt vascular trauma is more complicated than those of penetrating ones with a greater likelihood to compromise the arterial supply at an extensive zone. In addition, the vessel and surrounding structures following blunt trauma usually sustain more extensive injury, leading to severe destruction of the main and collateral blood supply. In such injuries, the management was more complicated and accordingly the amputation rates were usually higher in comparison with those with stab wounds.[2,8] As reported, multilevel arterial injuries due to blunt trauma may contribute to a 10-fold increased risk when compared with other injury patterns.[16] Consistent with previous reports [2,8,16], all the patients in this series were injured by blunt trauma. However, no significant differences in the mechanism of injury were seen among the three patterns.

Since multilevel arterial injuries are quite uncommon in clinical settings, misdiagnosis may readily occur as the clinician may concentrate on the highly suspected site and neglecting to recognize additional vascular injuries, resulting in high amputation rates and increased mortality. No related studies focusing on the multilevel arterial injuries were available in the literature. In the present study, the occurrence of missed diagnosis was 31.6%; of the three patterns, pattern B accounts for a much higher missed diagnosis rate of 70%, which means the extra vascular injury related to two isolated vascular injuries are more prone to be neglected; while it could be more easily diagnosed when occurring in two adjacent arteries, such as in pattern A and pattern C with missed diagnosis rates of only 20% and 15.4%, respectively. The severity of the trauma may also interfere with clinician’s ability to make a definitive diagnosis. Patients in pattern B suffered from a lower fracture occurrence rate of only 20% in comparison with 86.7% and 76.9% in pattern A and pattern C, respectively, misleading surgeons to making an imprudent diagnosis due to focusing on the lack of bony injuries.

The identification of multilevel arterial injuries is the prerequisite for a better outcome with a higher limb salvage rate. In this series, the amputation rate was only 11.5% (3/26) in patients with a definitive diagnosis, while it was approximately 5 times higher in those with a missed arterial injury. Of the three patterns, the amputation rate in pattern B (60%) was much higher than the other two patterns (13.3% and 15.4% in pattern A and III, respectively). This result seems not to be in line with the commonsense of an expected worse result when popliteal artery was involved. [6] However, it was closely correlated with the initial definitive diagnosis rate. It is conceivable that all the multilevel arterial injured cases with definitive diagnosis will be treated primarily with restoration of limb blood supply in a shorter time, which is vital in limb salvage and functional recovery as well.[16–18] Otherwise, secondary exploration is required for those who are misdiagnosed, putting the vulnerable tissues in peril of unsalvageable changes with potentially devastating consequences. Although the higher amputation rate in pattern B, which represents multilevel trauma of distant regions with a likelihood of a larger area of injured soft tissues, is probably due to a more extensive soft tissue trauma, it is conceivable that making a timely definite diagnosis is crucial in limb salvage no matter what level of artery injuries is located.

DUS is portable, cheap and non-invasive and it can be conveniently used for severely injured patients at the bedside. Although few studies specially focused on the use of DUS in extremity blunt vascular injuries, its accuracy when diagnosing blunt cervical vascular injury was found to be questionable. In addition, a high occurrence of missed injuries was also reported in some studies with a low sensitivity Therefore, some authors suggested that ultrasonography should not be advocated for suspected blunt cervical vascular injury. [19,20] In our practice, lower extremity arterial injuries can be detected easily by duplex ultrasonography with sensitivity of 100%; however, it is challenging to identify the existence of multilevel arterial injuries and its specificity was only 14.8%. Our findings also indicate that the values of DUS should be modestly considered in the diagnosis of blunt extremity arterial injuries. Arteriography and computed tomography angiography (CTA) have been utilized for the diagnosis of vascular injuries in patients suffering from vascular trauma in some studies with satisfactory outcomes.[21–25] Unfortunately, such examinations were not utilized in this series partly due to the concerns of high cost, technical availability, time consuming and inconvenience. Undoubtedly, if these work-ups were performed, the misdiagnosis rate would be significantly decreased. All the concerns neglecting the merit of these examinations should be reconsidered and a comprehensive evaluation of these techniques in the diagnosis of such cases is under further investigation in practice. But anyway, based on the widely accepted protocols in the management of extremity vascular injuries [26,27], more liberal use of arteriography should be considered in dealing with such cases.

For the treatment of vascular injuries, “although surgical technique affects outcome, results are primarily dependent on early detection of vascular injury followed by immediate treatment.” [28] The preoperative evaluation is valuable in diagnosis; however, more attention should be paid to intraoperative exploration in the management of blunt arterial injuries. Intraoperative arteriography is undoubtedly a very useful method to identify the exact vascular injuries with less trauma to the limbs[29]; however, to some extent, it is technical demanding and is restricted by device limitations. In contrast, surgical intervention may be more feasible in most hospitals of the developing countries. The extent of surgical exploration is suggested to be performed in a more aggressive way in comparison with the treatment of penetrating injuries and it is advisable to exceed the trauma zones. Sometimes tandem distal lesions can be treated by endovascular means using a vascular stent with no need of extensive exploration[30], but it has little merit in diagnosis. On the other hand, the routine judgment of vascular patency based on the blood reflux from the ends is not always reliable, especially around the knee area because of the abundant collateral circulations. Although a palpable dorsalis pedis artery pulse is a reliable indication for a successful repair, it is not uncommon that the absence of dorsalis pedis artery pulse doesn’t always mean that the vascular anastomosis has failed since patients sometimes are still in a state of shock with low blood pressure shortly after surgery. Nonetheless, after resuscitation with restoration of blood pressure, the dosalis pedis artery pulse should be palpated and easily detected by DUS. Therefore, postoperative monitoring is of great importance in order to identify a vascular crisis in a timely manner and to confirm the patency of a repaired artery.

The drawbacks of this study include: the demographic data are not detailed enough; the outcomes mainly concentrate on the limb salvage or amputation rate without regard to long-term function; other factors, such as the severity of trauma to additional structures and accompanying venous injuries, were just grossly documented, which may affect the outcomes and weaken the power of comparison among groups. The data of the Ankle and Brachial Index (ABI), one of the common parameters in evaluation of lower extremity arterial injures, were not collected in this study. Although it has little room in the differential diagnosis of multilevel arterial injuries, it may benefit the assessment of the patency of repaired arteries postoperatively. In addition, intraoperative angiograms were not use in this study, which may provide accurate information for definite diagnosis; the computed tomography angiography (CTA), as another essential modality of assessment in patients with suspected vascular injuries who lack the hard signs of vascular injury that mandate immediate surgical exploration, was also not carried out in this series. These techniques are especially important for the diagnosis and management of patients with suspected multi-level crush injuries featured in the present study. Therefore, our findings can only be interpreted with exclusion of the impact of these factors.

In conclusion, multilevel arterial injury of the lower extremity due to blunt trauma is limb-threatening and easily missed with potentially devastating consequences. The diagnosis of multilevel arterial injuries associated with femoral artery and posterior or anterior arteries is prone to be missed. DUS has low specificity in the detection of multilevel arterial injuries. A well-organized perioperative protocol, especially aggressive intraoperative exploration, is considered to be valuable in the definitive diagnosis of such injuries.

## Supporting Information

S1 Dataset Patients’ information. Injury patterns: (Zone I, femoral artery; Zone II, popliteal artery; Zone III, posterior and anterior tibial artery). A: Zone I + Zone II. B: Zone I + Zone III. C: Zone II + Zone III.
(XLS)
